# Residual Predictive Information Flow in the Tight Coupling Limit: Analytic Insights from a Minimalistic Model

**DOI:** 10.3390/e21101010

**Published:** 2019-10-17

**Authors:** Benjamin Wahl, Ulrike Feudel, Jaroslav Hlinka, Matthias Wächter, Joachim Peinke, Jan A. Freund

**Affiliations:** 1 Institute for Chemistry and Biology of the Marine Environment, University of Oldenburg, 26129 Oldenburg, Germany; ulrike.feudel@uni-oldenburg.de (U.F.); jan.freund@uni-oldenburg.de (J.A.F.); 2 ForWind-Center for Wind Energy Research, Institute of Physics, University of Oldenburg, 26129 Oldenburg, Germany; matthias.waechter@uni-oldenburg.de (M.W.); joachim.peinke@uni-oldenburg.de (J.P.); 3 Research Center Neurosensory Science, University of Oldenburg, 26129 Oldenburg, Germany; 4 Institute of Computer Science of the Czech Academy of Sciences, 18207 Prague, Czech Republic; hlinka@cs.cas.cz; 5 National Institute of Mental Health, 25067 Klecany, Czech Republic

**Keywords:** time series, information transfer, causality

## Abstract

In a coupled system, predictive information flows from the causing to the caused variable. The amount of transferred predictive information can be quantified through the use of transfer entropy or, for Gaussian variables, equivalently via Granger causality. It is natural to expect and has been repeatedly observed that a tight coupling does not permit to reconstruct a causal connection between causing and caused variables. Here, we show that for a model of interacting social groups, carried from the master equation to the Fokker–Planck level, a residual predictive information flow can remain for a pair of uni-directionally coupled variables even in the limit of infinite coupling strength. We trace this phenomenon back to the question of how the synchronizing force and the noise strength scale with the coupling strength. A simplified model description allows us to derive analytic expressions that fully elucidate the interplay between deterministic and stochastic model parts.

## 1. Introduction

In many scientific problems, causal interactions of different processes or process components are clear from a mechanistic understanding of the system dynamics and reflected by model formulation. In other situations, the interaction structure of a large and/or complex system, e.g., the assembly dynamics of a chemical system [1,2], of an ecological [3,4] or social community [5,6] or of neuronal populations [7,8], may not be clear in advance. Then, the empirical reconstruction of causal interactions from multivariate time series can be attempted using a suitable concept of causality. Since the seminal works of Wiener [9] and Granger [10], this reconstruction is based on the rationale that a causal interaction can be inferred from an increased prediction error when excluding the causing process component from the prediction of the caused component. Operationalizing this idea leads to Granger causality (GC), a measure commonly used to compare causal interactions of different process components. The quantification of a predictive information flow is also behind the definition of transfer entropy [11], which quantifies the predictive information transfer from the causing to the caused component. As shown by Barnett et al. [12], Granger causality and transfer entropy are equivalent for Gaussian variables.

From the outlined definition, it is plausible to expect that a stronger coupling will enhance the predictive information flow. On the other hand, it can be expected that an increasing coupling strength will eventually synchronize the caused with the causing variable. The approach to the synchronization manifold can be monitored by the synchronization index (phase locking value) or, for a linear dynamics, by the cross-correlation coefficient. Once the dynamics is tied to the synchronization manifold, a reconstruction is no longer possible because in a synchronous mode of motion leader and follower cannot be told apart—early reports of this insight [13,14] were followed by repeated observations of the phenomenon in the context of various directionality indices and synchronization measures (e.g., [15,16,17,18,19]), which underpins a generic phenomenon. Combining the synchronization triggered decline of directionality measures with the initial increase in the small coupling regime immediately suggests unimodal curve describing the relation between directionality and coupling strength, where any directionality index vanishes for zero (uncoupled) and infinite (tight coupling limit) coupling strength.

We note that, to the best of our knowledge, all reports of this phenomenon are solely based on numerical or empirical data and subsequent estimation of directionality indices. Moreover, directionality indices are mostly constructed as differences (asymmetries) between directed coupling indices, thus quantifying a two-way net directionality. Quite often estimators of the one-way directionality indices are plagued by a considerable bias and, in particular, non-zero values for uncoupled variables. Computing the net directionality index this bias may (or may not) be removed by forming differences. Therefore, an analytic treatment of a one-way directionality index vs. coupling strength relation for a tractable but generic dynamics seems desirable.

In the following, we address such a system dynamics that follows from a socio-dynamic model originally formulated in the framework of a master equation [20]. In the thermodynamic limit, the description is shifted to the level of a Fokker–Planck equation [21] with related drift and diffusion coefficients. The resulting expressions show that the coupling strength enters the diffusion matrix which means that random fluctuations scale up together with increasing coupling strength. Our analytic derivation leads to the interesting result that even in the tight coupling limit a residual directionality remains. Since the resultant dynamics can effectively be mapped to a vector-autoregressive process (linear stochastic process with Gaussian variables), the residual Granger causality can be interpreted as a residual predictive information flow remaining for tight coupling. Although we use a specific model at the outset of our presentation, we argue that the finding of a residual GC is a generic effect.

## 2. Granger Causality in a Nutshell

To set the stage, we briefly recall how GC is defined in the framework of a (two-dimensional) vector autoregressive process VAR[*∞*] (of order infinity). In this case, two time series are fitted by the following full model

(1) $$\begin{array}{cc} X_{1,t} & =\sum_{j=1}^{\infty }a_{j}X_{1,t-j}+\sum_{j=1}^{\infty }b_{j}X_{2,t-j}+\epsilon_{t},\\ X_{2,t} & =\sum_{j=1}^{\infty }c_{j}X_{1,t-j}+\sum_{j=1}^{\infty }d_{j}X_{2,t-j}+\eta_{t} \end{array}$$

with white noise covariance matrix

(2) $$\begin{array}{cc} \Sigma & =\left(\begin{array}{cc} \Sigma_{11} & \Sigma_{12}\\ \Sigma_{21} & \Sigma_{22} \end{array}\right)=\left(\begin{array}{cc} \mathrm{var}(\epsilon_{t}) & \mathrm{cov}(\epsilon_{t},\eta_{t})\\ \mathrm{cov}(\epsilon_{t},\eta_{t}) & \mathrm{var}(\eta_{t}) \end{array}\right). \end{array}$$

In describing stable (non-divergent) time series by a VAR, the dynamical coefficients $a_{j},b_{j},c_{j}$ and $d_{j}$ will obey a stationarity condition [22].

The prediction error of $X_{2}$, using both its own past *and* the past of $X_{1}$, is quantified by $\Sigma_{22}$, the variance of $\eta_{t}$. Predicting $X_{2}$ *only* from its own past means to use a reduced univariate autoregressive processes description for the second time series

(3) $$\begin{array}{ccc} X_{2,t} & = & \sum_{j=1}^{\infty }{\tilde{d}}_{rj}X_{2,t-j}+{\tilde{\eta }}_{t},\;\mathrm{var}\left({\tilde{\eta }}_{t}\right)=\Xi_{2}. \end{array}$$

In passing, we note that the parameters of this reduced model are not fitted independently but derived from Equation (1) for example through a spectral factorization [23]. Moreover, based on a state-space formulation of GC, a closed form involving partial covariances can be obtained for finite order vector-autoregressive models [24].

Now, the prediction error of $X_{2}$ without inclusion of $X_{1}$ is quantified by $\Xi_{2}$, the variance of ${\tilde{\eta }}_{t}$. Since in this step no information is added (but rather removed), $\Xi_{2}$ will never be smaller than $\Sigma_{22}$, guaranteeing that Granger causality GC (from $X_{1}\to X_{2}$) defined as the quantity $\log(\frac{\Xi_{2}}{\Sigma_{22}})$ is non-negative.

VAR processes belong to the model class of time discrete linear stochastic processes. In a recent work, we extended the concept of GC to diffusion processes which constitute a special class of time continuous stochastic processes. Diffusion processes can be formulated either via a Langevin type stochastic differential equation system or via the related Fokker–Planck equation (FPE) [25]. In the framework of a local linearization of the dynamics and a stroboscopic map (i.e., the temporal integration of the dynamic flow over a time increment Δt), this allows establishing a connection between drift and diffusion coefficients of the FPE and a local GC. Averaging the local GC with respect to the invariant measure yields a global GC for the original diffusion process. For details, we refer the reader to our recent publication [26].

## 3. Weidlich’s Socio-Dynamic Model

Here, we apply this connection to a stochastic socio-dynamics model proposed by Weidlich [20] to describe opinion formation in a system with two groups, *leaders* (Group 1 comprising $n_{1}$ members) and *followers* (Group 2 with $n_{2}$ members). Individuals of each group i=1,2 can adopt one of two opinions j=1,2. Opinion diversity across the community is coded by variables $n_{i,j}$ specifying the number of individuals of group *i* sharing opinion *j*. Since it is assumed that everybody has an opinion, $n_{i,1}+n_{i,2}=n_{i}$. Eventually, the system is considered in the “thermodynamic limit” $n_{1},n_{2}\to \infty$ with $n_{1}/n_{2}=\epsilon ,$ which suggests a change to quasi-continuous variables

(4) $$0\leq y_{i,j}:=\frac{n_{i,j}}{n_{i}}\leq 1\;\mathrm{with}\;y_{i,1}+y_{i,2}=1\;.$$

Members of group *i* change their opinion from *j* to $j^{\prime }$ with a transition rate $p_{j\to j^{\prime }}^{\left(i\right)}$ that may depend on the present opinion configuration $\left\{y_{\alpha ,\beta }\right\}$ (with α,β=1,2). In line with Weidlich, we employ the following expressions

(5) $$\begin{array}{c} p_{1\to 2}^{\left(1\right)}=\frac{3}{4}p,\;p_{2\to 1}^{\left(1\right)}=\frac{1}{4}p,\;p_{1\to 2}^{\left(2\right)}=\frac{1}{4}p+\mu p\cdot y_{1,2},\;p_{2\to 1}^{\left(2\right)}=\frac{3}{4}p+\mu p\cdot y_{1,1} \end{array}$$

with a straightforward interpretation: without coupling (μ=0) leaders and followers have a diametrical preference for one opinion over the alternative one; while leaders flip opinion without reference to the population of followers ($y_{2,j}$), with increasing coupling followers enhance the rate of switching their opinion to the opinion populated by leaders ($y_{1,j}$). This means that followers are uni-directionally forced by leaders with the coupling strength scaled by the parameter μ.

These rates enter a master equation describing the evolution of a statistical ensemble of interacting social groups. After another change of variables $x_{i}=y_{i,2}-y_{i,1}$, reflecting directed imbalance and opinion diversity within group *i*, the related Fokker–Planck equation can be written down [20]. It is completely specified by its drift vector

(6) $$\begin{array}{cc} D^{\left(1\right)} & =-p\left(\begin{array}{c} x_{1}-\frac{1}{2}\\ x_{2}+\frac{1}{2}+\mu \cdot \left(x_{2}-x_{1}\right) \end{array}\right) \end{array}$$

and its diffusion matrix

(7) $$\begin{array}{c} D_{11}^{\left(2\right)}=\frac{p}{n_{1}}\left(2-x_{1}\right),\;D_{22}^{\left(2\right)}=\frac{p}{n_{2}}\left(2+x_{2}+2\mu \left(1-x_{1}x_{2}\right)\right),\;D_{12}^{\left(2\right)}=D_{21}^{\left(2\right)}=0\;. \end{array}$$

Notice that the diffusion matrix is positive definite since $-1\leq x_{1},x_{2}\leq 1$. The inverse scaling with $n_{1}$ and $n_{2}$, respectively, means that diffusion vanishes in the “thermodynamic limit” $n_{1},n_{2}\to \infty$ (with $\epsilon =n_{1}/n_{2}$). Moreover, since the parameter *p* (controlling the intrinsic opinion flip rate) scales both drift vector and diffusion matrix in the same way, it may well be absorbed in a reparameterization of time. Finally, we point out that in the step from the master equation to the FPE the coupling parameter μ does not only enter the drift vector but also the diffusion matrix.

## 4. Simulation Results

**Figure 1:**
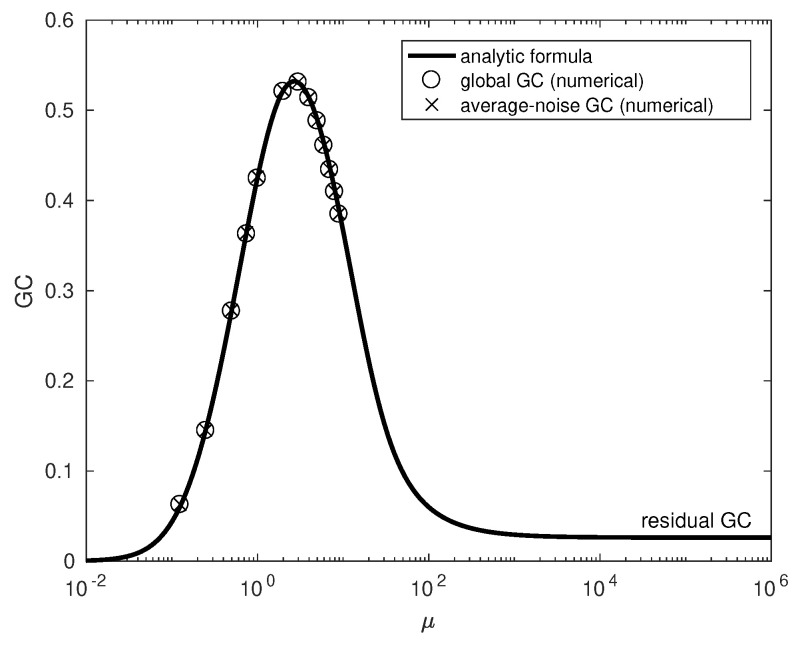
Dependence of Granger causality GC on the coupling parameter μ: circles, numerical simulation results of the Langevin equation based on drift and diffusion coefficients (Equations (6) and (7), respectively); crosses, GC of the global VAR(1) (Equation (8)) with average-noise; solid line, analytic GC (Equation (20)) of the minimalistic model.

Being equipped with drift vector and diffusion matrix, we can compute the GC of the socio-dynamic diffusion process (as outlined in [26]) for various values of the coupling parameter μ. This means we compute GCs locally for subsampled vector-Ornstein–Uhlenbeck processes with local additive noise; global GC is then obtained as the statistical average of local GCs with respect to a stationary distribution estimated from the simulation (1,000,000 data points for each μ value). Additionally, we compute the GC of a global vector-Ornstein–Uhlenbeck process with constant average-noise. The results are plotted with circle and crosses, respectively, in Figure 1.

As expected, for μ=0, the GC is zero because in this uncoupled situation inclusion of $x_{1}$ cannot reduce the prediction error of $x_{2}$. With increasing coupling, parameter μ GC initially increases up to a maximum around μ=3 before it decreases again. Surprisingly, we find that a linearized dynamics with average-noise (notice that the only non-linearity $∼x_{1}x_{2}$ resides in $D_{22}^{\left(2\right)}$) with state space averaged additive noise gives rise to the same values (plotted in Figure 1 with crosses). Therefore, the non-monotonic characteristic of GC requires neither a non-linear dynamics nor multiplicative noise.

## 5. Analytic Description

To explain the non-monotonic behavior of the GC, we connect the diffusion process specified by Equations (6) and (7) with a VAR(1), i.e., vector-autoregressive process of first order. The general connection is elaborated in [27] and proceeds via the VAR(1) written as

(8) $$X_{t}=AX_{t-1}+\theta_{t}$$

where

(9) $$\begin{array}{c} A=\exp\left[\left(\begin{array}{cc} -1 & \;0\\ \mu & \;-(1+\mu ) \end{array}\right)p\Delta t\right]=e^{-p\Delta t}\left(\begin{array}{cc} 1 & \;0\\ 1-e^{-\mu p\Delta t} & \;e^{-\mu p\Delta t} \end{array}\right)\; \end{array}$$

and $\theta_{t}$ is a two-component vector of zero-mean Gaussian white noise residuals with a covariance matrix Σ given by the following matrix integral

(10) $$\Sigma =\int_{0}^{\Delta t}e^{\Gamma t}\left(\begin{array}{cc} {\hat{D}}_{11}^{\left(2\right)} & \;0\\ 0 & \;{\hat{D}}_{22}^{\left(2\right)} \end{array}\right)e^{\Gamma^{†}t}dt\;,$$

where Γ is the Jacobian matrix of the diffusion process, i.e., of its drift part.

The above connection is derived for the standard vector-Ornstein-Uhlenbeck process with additive noise, which corresponds to a diffusion matrix ${\hat{D}}_{ij}^{\left(2\right)}$ that is constant in state space. By contrast, in Weidlich’s model we see that the diffusion coefficients $D_{11}^{\left(2\right)}$ and $D_{22}^{\left(2\right)}$ (cf. Equation(7)) vary with $x_{1}$ and $x_{2}$. Since the gradients scale with the inverse of $n_{1}$ and $n_{2}$, respectively, for rather large $n_{1},n_{2}$, the noise is weakly multiplicative [26]. Therefore, we may consider $\Sigma (x_{1},x_{2})$ as a local covariance matrix. A rigorous derivation would now strive to compute local GC maps and perform a subsequent average over state space (with respect to the stationary probability distribution, essentially a bivariate Gaussian). This route seems feasible but resultant expressions appear far from being handy. Therefore, we approximate state dependent diffusion coefficients by substituting mean values for state variables, i.e.,

(11) $$D_{ij}^{\left(2\right)}\left(x_{1},x_{2}\right)\to D_{ij}^{\left(2\right)}\left(\langle x_{1}\rangle ,\langle x_{2}\rangle \right)\;.$$

The mean values can be read from Equation (6)

(12) $$\langle x_{1}\rangle =\frac{1}{2}\;\mathrm{and}\;\langle x_{2}\rangle =\frac{1}{2}\frac{\mu -1}{\mu +1}$$

with the consequence that we approximate

(13) $$\begin{array}{c} \sigma_{1}^{2}:=\langle D_{11}^{\left(2\right)}\left(x_{1},x_{2}\right)\rangle \approx \frac{p}{n_{1}}\frac{3}{2},\;\sigma_{2}^{2}\left(\mu \right):=\langle D_{22}^{\left(2\right)}\left(x_{1},x_{2}\right)\rangle \approx \frac{p}{n_{2}}\frac{1}{2}\frac{3\mu^{2}+10\mu +3}{\mu +1}\;. \end{array}$$

In line with these simplifications, we now consider the case of a VAR(1) with the following parameterization

(14) $$\begin{array}{c} X_{t}=\left(\begin{array}{c} X_{1,t}\\ X_{2,t} \end{array}\right)=\left(\begin{array}{cc} b & \;0\\ c & \;a \end{array}\right)\left(\begin{array}{c} X_{1,t-1}\\ X_{2,t-1} \end{array}\right)+\left(\begin{array}{c} \theta_{1,t}\\ \theta_{2,t} \end{array}\right), \end{array}$$

where the parameters a,b,c are to be identified with the entries in Equation (9), i.e.,

(15) $$\begin{array}{c} a=e^{-(1+\mu )h},\;b=e^{-h},\;c=e^{-h}-e^{-(1+\mu )h} \end{array}$$

where we have used the abbreviation h=pΔt.

The covariance matrix of residuals is obtained by inserting $\sigma_{1}^{2}$ and $\sigma_{2}^{2}\left(\mu \right)$ of Equation (13) into Equation (10). The integration can be performed analytically and leads to the following expressions

(16) $$\begin{array}{c} \Sigma_{11}=\frac{\sigma_{1}^{2}}{2p}\left(1-e^{-2h}\right),\;\Sigma_{12}=\Sigma_{21}=\frac{\sigma_{1}^{2}}{2p}\frac{\mu \left(1-e^{-2h}\right)-2\left(e^{-2h}-e^{-(2+\mu )h}\right)}{\mu +2}\\ \Sigma_{22}=\frac{\sigma_{2}^{2}\left(\mu \right)}{2p}\frac{1-e^{-2(1+\mu )h}}{\mu +1}+\frac{\sigma_{1}^{2}}{2p}\times \\ \times \frac{\mu^{2}\left(1-e^{-2h}\right)-\mu \left(3e^{-2h}-4e^{-(2+\mu )h}+e^{-2(1+\mu )h}\right)-2\left(e^{-2h}-2e^{-(2+\mu )h}+e^{-2(1+\mu )h}\right)}{\mu^{2}+3\mu +2}\;. \end{array}$$

Notice that

(17) $$\lim_{\mu \to \infty }\Sigma =\left(\begin{array}{cc} \Sigma_{11} & \;\Sigma_{11}\\ \Sigma_{11} & \;\left[1+\frac{n_{1}/n_{2}}{1-e^{-2h}}\right]\Sigma_{11}\; \end{array}\right)\;.$$

With $\eta :=\frac{n_{1}/n_{2}}{2(1-e^{-2h})}∼\epsilon$ being small (since $n_{1}\ll n_{2}$ and h≪1), this matrix has the eigenvalues

(18) $$\begin{array}{c} \lambda_{1,2}=\left(1+\eta \pm \sqrt{1+\eta^{2}}\right)\Sigma_{11}=\{\begin{array}{c} \left[2+\eta +O(\eta^{2})\right]\Sigma_{11}\\ \left[\eta +O(\eta^{2})\right]\Sigma_{11} \end{array}\;, \end{array}$$

which shows that even in the tight coupling limit the covariance ellipsoid of residuals possesses two finite semi-axes.

Equation (14) defines a VAR(1) that was recently considered by Barnett and Seth as a minimal model that allows deriving an analytic expression for GC that can be found in [28] and was restricted to the special case that Σ was the identity matrix, i.e., $\theta_{1,t}$ and $\theta_{2,t}$ were uncorrelated ($\Sigma_{12}=\Sigma_{21}=0$) and both with unit variance ($\Sigma_{11}=\Sigma_{22}=1$). Relaxing these restrictions and following the same steps outlined by Barnett and Seth we can derive an explicit formula for GC that is structurally identical to Barnett’s formula even for the most general, bivariate VAR(1) process in Equation (8) with bidirectional coupling ($A_{12}=d\neq 0$)

(19) $$\begin{array}{ccc} \;GC & = & \frac{1}{\pi }\int_{0}^{\pi }\ln\left(1+\frac{c^{\prime 2}}{1-2b^{\prime }\cos\lambda +b^{\prime 2}}\right)\;d\lambda \end{array}$$

(20) $$\begin{array}{cc} = & \ln\left(\frac{1+b^{\prime 2}+c^{\prime 2}+\sqrt{{\left(1+b^{\prime 2}+c^{\prime 2}\right)}^{2}-4b^{\prime 2}}}{2}\right) \end{array}$$

but with the replacement

(21) $$\begin{array}{c} b^{\prime }=b-\frac{\Sigma_{12}}{\Sigma_{22}}c,\;c^{\prime }=\frac{\sqrt{\det\Sigma }}{\Sigma_{22}}c\;. \end{array}$$

Through Equations (20) and (21) in conjunction with Equation (15), we have everything at hand to evaluate the dependence of GC on the coupling parameter μ. Note that the GC does not depend on the parameter *a*, and for a fixed covariance matrix is a monotonically increasing function of *c* growing to infinity; indeed, in a simple autoregressive model, the parameter *c* would be naturally considered (a monotonous function of) the coupling strength μ. However, in our example, the dependence of the c′ on coupling parameter μ is more complicated, as it also affects the covariance matrix. Moreover, the dependence is such that there is a delicate balance for infinite coupling strength μ between the synchronizing influence of the driver and the diffusion term leading to the finite residual GC. The result is plotted in Figure 1 (with solid line) and shows that the analytic formula perfectly matches the numerical values (plotted with circles and crosses) extracted from the simulation. The matching of our analytical results, based on Equation (11), and global GC is surprising but fortunate. The coincidence of global GC and average-noise GC, on the other hand, is not surprising since, for weakly multiplicative noise, the latter is in line with our analytic approach.

The analytic formula allows considering the asymptotic limit μ→∞, which is given by inserting

(22) $$\begin{array}{c} \lim_{\mu \to \infty }b^{\prime }=\frac{\epsilon e^{-h}}{1+\epsilon -e^{-2h}},\;\lim_{\mu \to \infty }c^{\prime }=\frac{\sqrt{\epsilon e^{-2h}\left(1-e^{-2h}\right)}}{1+\epsilon -e^{-2h}} \end{array}$$

into Barnett’s formula (Equation (20)). Interestingly, $n_{1}$ and $n_{2}$ again enter this expression only through the ratio $\epsilon =n_{1}/n_{2}$. For the chosen parameters, the resulting asymptotic value $\lim_{\mu \to \infty }$GC is approximately 0.027, which is larger than zero. As the scaling with ϵ∼η indicates, the asymptotic residual GC is a consequence of the residual variance in the direction of the second semi-axes of the covariance ellipsoid in Equation (18). Residual fluctuations remaining in the tight coupling limit enable an improvement of prediction.

**Figure 2:**
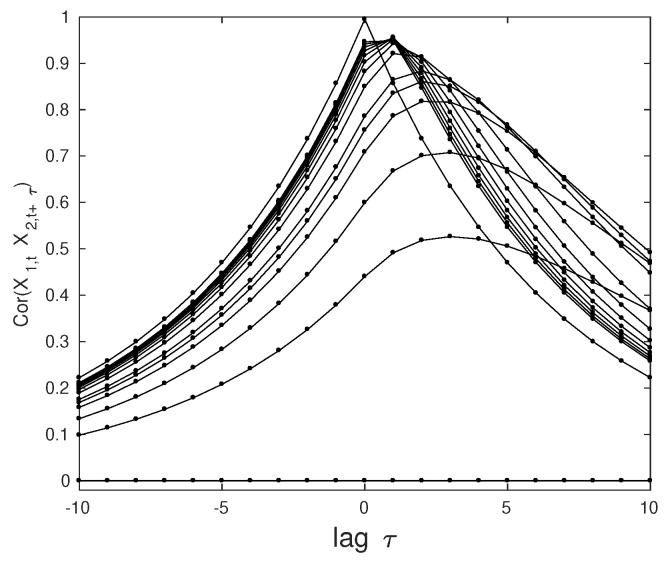
Cross-correlations for couplings $\mu =0,\frac{1}{8},\frac{1}{4},\frac{1}{2},\frac{3}{4},1,\ldots ,9,\infty$ (bottom to top for all non-positive lags) and various lags τ.

These residual fluctuations reflect the fact that even for infinite coupling strength synchronization is not complete. To show this, we compute the cross-correlation function by solving the Yule–Walker equations [22]. As can be seen in Figure 2, the maxima of the curves rise with increasing coupling strength while the related lag approaches zero (in discrete steps) reflecting instantaneous response of the caused variable. However, our analytic treatment reveals that even in the tight coupling limit μ→∞ the maximal cross-correlation is 0.995 and not one; the reason is, again, the balance between the synchronizing force (exponential relaxation) and the increasing noise strength $\sigma_{22}\left(\mu \right)$. We note that this value is not plagued by estimation errors.

The analytic approach also puts us in the position to sort out how the different system properties contribute to the formation of a maximum in the GC. In particular, we can investigate the effect of coupling dependent diffusivity through replacing $\sigma_{2}^{2}\left(\mu \right)$ (cf. Equation (13)) by the constant value $\sigma_{2}^{2}\left(\mu =0\right)=\epsilon \sigma_{1}^{2}$. Through these investigations, we arrive at the following conclusions:

- For uncoupled systems (μ=0), GC is zero because $c^{\prime }=0$.
- The initial increase of GC is caused by the exponential increase of *c* towards *b* with increasing coupling *c* (cf. Equation (15)). Residual variance of the process component $X_{2}$ is reduced via information transferred from the driver $X_{1}$.
- Simultaneously with the information transferred from the driver intrinsic fluctuations, modeled by increasing diffusivity $\sigma_{2}^{2}\left(\mu \right)$, scale up (asymptotically ∼μ). Nevertheless, the residual variance $\Sigma_{22}$ approaches a constant because tighter coupling to $X_{1}$ (as expressed by the denominator μ+1 in Equation (16)) compensates increasing intrinsic noise. In the limit μ→∞, the residual variance $\Sigma_{22}$ is the residual variance $\Sigma_{11}$ of the driver plus an extra term resulting from the balance between intrinsic noise and tight coupling, i.e., $\lim_{\mu \to \infty }\Sigma_{22}=\left[1+\frac{n_{1}/n_{2}}{1-e^{-2h}}\right]\Sigma_{11}$
- From the imbalance of diffusivities follows the ratio
  (23) $$\frac{\Sigma_{22}\left(\mu \to \infty \right)}{\Sigma_{22}\left(\mu =0\right)}=\frac{1}{\epsilon }\left(1+\frac{\epsilon }{1-e^{-2h}}\right)∼\frac{1}{\epsilon }$$
  which means that for ϵ=0.01 the residual variance $\Sigma_{22}$ is increasing roughly by the factor 100. As can be read from Equation (21), this tends to reduce $c^{\prime 2}$ with increasing μ. From the opposing trends - $c^{2}$ increases with μ while $1/\Sigma_{22}$ decreases with increasing μ - the peak results as the point where the dominance of both trends changes. We note that analytic expressions can be derived since we have all ingredients at hand, however, explicit formulas are quite lengthy and do not provide deeper insights. In all cases considered, we found that $\sqrt{\det\Sigma }$ varied only mildly with μ and the variation of $b^{\prime 2}$ is of minor importance for Barnett’s formula (Equation (20)).

## 6. Discussion

The increase of GC with μ is a natural consequence of enhanced information transfer with tighter coupling to the driver. The asymptotic decrease and the formation of a maximum, on the other hand, follow from the fact that the caused variable $X_{2}$ asymptotically adopts the residual variance of the driver which is two orders larger because $n_{2}/n_{1}=\epsilon^{-1}=100$, meaning that “every leader has one hundred followers”. This allows predicting that no such maximum can be found if $n_{1}\approx n_{2}$ or for $n_{1}>n_{2}$, i.e., if the leader group is of comparable or even larger size than the follower group—a statement that we can confirm through our analytic analysis. It is interesting to note that the asymptotic residual GC, i.e., an improvement of prediction in the tight-coupling limit, remains even in the thermodynamic limit $n_{1},n_{2}\to \infty$, i.e., when the diffusion constants approach zero, provided $\epsilon =n_{1}/n_{2}>0$. This results from the scaling behavior of the variance $\Sigma_{22}$.

The question whether the found residual GC is a property of the specific socio-dynamic model or a generic property of a larger class of systems can be answered in the following way: our results follow from a minimalistic linear system (vector-Ornstein–Uhlenbeck process or VAR(1)), which means it is not a fancy effect of non-linear dynamics. In this setup, the rGC results from the linear asymptotic scaling behavior of $\sigma_{2}^{2}\left(\mu \right)$ (Equation (13)), which balances the μ in the denominator of the first term in Equation (16) resulting from integrating the exponentials (in Equation (10)) and reflecting the faster relaxation with increasing coupling strength. The question as to which other systems might give rise to a residual GC thus leads back to the linear scaling of the diffusion coefficient $D_{22}^{\left(2\right)}\left(\mu \right)$. As the derivation in Weidlich’s original paper [20] shows, this is a consequence of the ansatz of coupling groups via a coupling parameter that enters the flipping rates (Equation (5)) additively and linearly. Therefore, we conclude that a residual GC will not be restricted to the specific details of Weidlich’s model but be a quite generic aspect of a stochastic dynamics described by a similar master equation.

The observed non-monotonic behavior of GC with coupling strength does not rely on non-linear dynamics or multiplicative noise. A peak of the information flow was pointed out and a finite value of the information flow, at large couplings, was also reported in [29]. However, it is interesting to consider how our insights can be transferred to a general diffusion process. Our conclusions drawn from the minimalistic system (see Equation (14) with Equation (15)), i.e., a linear dynamics with additive noise, can be carried over to the class of nonlinear diffusion processes with weakly multiplicative noise in the framework of a local linearization generating a GC map in state space. Subsequent averaging with respect to the invariant measure on the attractor leads to the global GC of the system, a concept that we put forward in a recent publication [26]. In this way, the global GC vs. coupling relation will be computable and the resultant curve will depend on the details of the considered system. The question whether a non-monotonic GC vs. coupling relation can result for a system with diffusion constants that do *not* vary with coupling strength remains open and will be dealt with in a forthcoming publication.

Finally, one may ask whether the non-monotonic GC curve plays any role in natural systems. We speculate that coupling enhanced predictability might be useful for the performance of cognitive functions and that certain brain regions are tuned to optimal synaptic coupling. In this context, a scale up of effects through coupling large neuronal ensembles can also be imagined.

## 7. Summary

We analytically investigated the non-monotonic behavior of Granger causality, defined on a socio-dynamic model of leaders and followers, with respect to coupling strength. Our derivations finally substantiate frequent observations of this phenomenon for various directionality indices and agree with the common sense that synchronization impedes a reconstruction of causal relationships. To make this point, it was necessary to extend Granger causality of Barnett’s minimal model to a general vector-autoregressive process of order one. Fortunately, we were able to demonstrate that a tight coupling in the master equation can map not only to the dynamics but also to the noise and therefore does not automatically imply synchronization and a vanishing Granger causality.
